# Co-targeting IGF-1R and c-kit: synergistic inhibition of proliferation and induction of apoptosis in H 209 small cell lung cancer cells

**DOI:** 10.1038/sj.bjc.6601682

**Published:** 2004-04-20

**Authors:** A Camirand, M Pollak

**Affiliations:** 1 Lady Davis Institute for Medical Research and Department of Oncology, McGill University, Montréal, QC, Canada

**Keywords:** IGF-1R, c-kit, co-targeting, small cell lung cancer, synergy

## Abstract

Most small cell lung cancers (SCLC) coexpress the c-kit protein tyrosine receptor kinase and its ligand stem cell factor, resulting in an autocrine loop. As SCLC growth is also driven by insulin-like growth factor-1 receptor (IGF-1R) signalling, tyrphostins AG 1024 and 1296 (inhibitors of IGF-1R and c-kit activity, respectively) were used to co-target these receptors in H 209 SCLC cells. Combination treatment caused synergy in proliferation inhibition and in apoptosis induction, and also enhanced reduction in phosphorylation of Erk1/Erk2, suggesting that co-targeting IGF-1R and c-kit in SCLC may be more effective than single-agent therapies.

The signalling activity of receptor protein tyrosine kinases (PTKs) tightly controls crucial cellular processes such as apoptosis, differentiation and proliferation, and it is therefore not surprising that deregulation of these molecules often leads to neoplastic progression. The involvement of PTKs in the pathology of many cancers has brought attention to these receptors as potential targets for therapeutic intervention (Morin, 2000; Shawver *et al*, 2002; Dancey and Sausville, 2003). Some neoplastic conditions result from excessive activity of a single PTK (Bcr-Abl in chronic myeloid leukaemia (Melo, 1996), c-kit or PDGFRA in gastrointestinal stromal cell tumours (Hirota *et al*, 1998; Heinrich *et al*, 2003)), and are effectively treated using the PTK inhibitor Imatinib (STI 571, Gleevec) (Druker *et al*, 2001; van Oosterom *et al*, 2001). However, most cancers involve dysfunction of more than one PTK and crosstalk between their downstream signalling pathways. A therapeutic approach to address this issue involves co-targeting different PTKs (Wu *et al*, 1995; Ye *et al*, 1999; Moasser *et al*, 2001; Moulder *et al*, 2001; Lu *et al*, 2001; Chakravarti *et al*, 2002; Totpal *et al*, 2002; Camirand *et al*, 2002). For co-targeting approaches to be useful, careful consideration must be given to the choice of PTKs to be simultaneously blocked (Dancey and Sausville, 2003).

Small cell lung cancer (SCLC) is an aggressive cancer with poor prognosis, which readily metastasises outside the chest, and has a median survival of approximately 9 months (Chute *et al*, 1999). SCLC has been treated with traditional combination chemotherapy since the 1970s, but less than 3% of patients with extensive-stage SCLC survive more than 3 years (Chute *et al*, 1999). The molecular pathology of more than 70% of SCLC cases involves autocrine stimulation due to the coexpression of c-kit and its ligand stem cell factor (SCF) (Hibi *et al*, 1991), and high c-kit expression level is associated with a poor prognosis (Micke *et al*, 2003). Imatinib (which targets Bcr-Abl, c-kit, and PDGFR (Morin, 2000)), tyrphostin AG 1296 (which inhibits c-kit and PDGFR (Kovalenko *et al*, 1994)) and indolinones SU 5416 and SU6597 (inhibitors of c-kit and related kinases (Krystal *et al*, 2001)) reduce SCLC proliferation *in vitro* (Krystal *et al*, 1997, 2000, 2001; Wang *et al*, 2000; Kijima *et al*, 2002). Phase I and II clinical trials with Imatinib are ongoing for SCLC.

Another growth pathway known to sustain proliferation in SCLC is the IGF-1R signalling cascade (Nakanishi *et al*, 1988; Macaulay *et al*, 1990). In many cancers, IGF-1R signalling leads to activation of PI-3 K and MAPK pathways, stimulates proliferation, promotes angiogenesis and metastasis, and inhibits apoptosis (Khandwala *et al*, 2000; Pollak, 2000; Yu and Rohan, 2000; Wang and Sun, 2002). Preclinical work has demonstrated that IGF-1R could be used as a successful co-target with EGFR (Chakravarti *et al*, 2002) and HER2/erbB2 (Lu *et al*, 2001; Camirand *et al*, 2002), and in the latter study, synergistic inhibition of proliferation was observed in co-targeted breast cancer cells. The availability of several novel inhibitors of IGF-1R signalling (Ludwig *et al*, 2003; Garcia-Echeverria *et al*, 2003; Mitsiades *et al*, 2003) implies that clinical exploitation of any synergism between co-targeting of c-kit and IGF-1R will be practical.

In this study, we use tyrphostins AG 1024 (IGF-1R inhibitor) and AG 1296 (PDGFR and c-kit inhibitor) to inhibit PTK activity in H 209 SCLC cells which present no PDGFR receptors. We demonstrate that combination treatment causes synergy in inhibition of proliferation and in apoptosis induction in these cells, and we speculate that co-targeting the IGF-1R and c-kit signalling pathways is potentially a more effective approach for this system than single-agent therapies.

## MATERIALS AND METHODS

### Cell culture and proliferation assays

H 209 SCLC cells were obtained from ATCC (Manassas, VA, USA) and cultured at 37°C with 5% CO_2_ in RPMI 1640 medium with 10% foetal bovine serum (FBS) (InVitrogen, Gaithersburg, MD, USA) except in growth inhibition assays where the FBS supplement was reduced to 1%. Cell proliferation was measured with the Alamar Blue dye reduction method (Biosource International, Camarilo, CA, USA), which is optimal for cells growing in anchorage-independent conditions. Tests were conducted with 10^4^ cells well^−1^ in 200 *μ*l media in 96-well plates (edge wells were not used, but filled with 200 *μ*l PBS) and three replicates per dose combination were used for each experiment. Stock solutions (10 mM) in DMSO of tyrphostins AG 1024 and AG 1296 (Calbiochem, San Diego, CA, USA) were kept at −20°C and diluted in 1% medium just before use. DMSO concentration in final culture was kept below 0.2% (v v^−1^). All procedures involving tyrphostins were conducted in low light intensity.

### Detection of surface receptors

H 209 cells were collected by centrifugation and washed with FACS buffer 4°C (PBS containing 3% FBS and 0.02% NaN_3_). Approximately 10^6^ cells were stained with phycoerythrin (PE)-conjugated anti-IGF-1R, anti-PDGFR*α*, or PDGFR*β* antibodies (BD Pharmingen, San Diego, CA), or with fluorescin (FITC)-conjugated anti-c-kit (CD 117) antibody (Santa Cruz Biotechnology, Santa Cruz, CA, USA). Cells were incubated with the antibodies for 30 min at 4°C in the dark, washed twice, and resuspended in FACS buffer. Analysis was conducted for 20 000 cells using a FACSCalibur flow cytometer (BD Biosciences, Burlington, MA, USA) with CellQuest software (BD Biosciences Immunocytometry Systems, Franklin Lakes, NJ, USA). Normal mouse IgG_1_ (Santa Cruz Biotechnologies) was used for isotype determination.

### Cell cycle analysis

Approximately 10^6^ cells were collected by centrifugation, washed twice in PBS and fixed in ice-cold 70% ethanol for 1 h, then stained for 30 min in the dark at 37°C in 1 ml propidium iodide (PI) staining buffer (0.1% Triton X-100, 0.1 mM EDTA pH 7.4 in PBS, to which 50 *μ*g ml^−1^ PI and 0.05 mg RNAse were added just before use). DNA staining was determined by flow cytometry on a FACSCalibur using CellQuest software (see previous section).

### Induction of apoptosis

Approximately 10^6^ cells were collected by centrifugation and washed in PBS, then stained with annexin-V-FITC and with PI using the ApoTarget kit (Biosource International, Camarillo, CA, USA). Analysis was conducted by flow cytometry on a FACSCalibur using CellQuest software (see receptor section above).

### Western blots

H 209 cells (approx. 3 million) were treated with various doses of tyrphostins or vehicle for a period of 72 h. Cells were lysed in nondenaturing buffer (1% Nonidet NP-40, 20 mM Tris-Cl pH 8.0, 0.5 mM Na orthovanadate pH 9.0 and proteinase inhibitors (Roche, Mannheim, Germany), and freeze-thawed 3 times. Insoluble material was removed by centrifugation, and 50 *μ*g aliquots of the supernatant were separated on 12% polyacrylamide gels. After transfer to TransBlot nitrocellulose membranes (BioRad, Hercules, CA, USA), the proteins were reacted O/N with the following primary antibodies: anti-Akt, anti-phospho-Akt (ser 473), anti-Erk1/Erk2 (p44/42), and anti-phospho Erk1/Erk2 (Thr202/Tyr204) (Cell Signalling, Beverly, MA, USA) at 1 : 1000 dilution, then 1 h with 1 : 2000 horseradish peroxidase-conjugated anti-rabbit immunoglobulin G (Pharmacia-Amersham, Piscataway, MJ, USA). Blots were reacted with the ECL chemoluminescence kit (Pharmacia-Amersham, Piscataway, NJ, USA) and exposed to X-OMAT LS film (Kodak, Rochester, NJ, USA). Densitometry analysis was conducted using the Scion Image software (Scion Corp., Frederick, MD, USA).

## RESULTS

**Figure 1:**
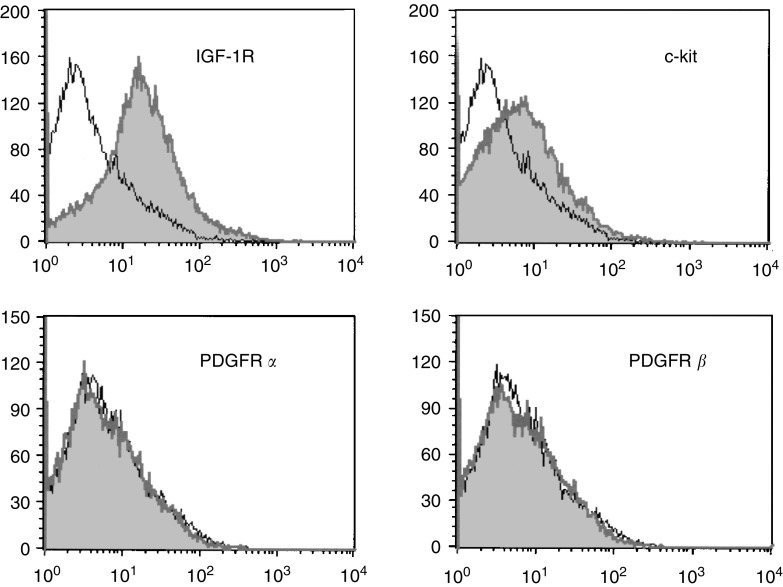
Surface expression of IGF-1R, c-kit, PDGFR *α*, and PDGFR *β* on H 209 SCLC cells. H 209 cells collected by centrifugation were stained with phycoerythrin (PE)-conjugated anti-IGF-1R, anti-PDGFR *α*, anti-PDGFR *β* antibodies, or fluorescein (FITC)-conjugated anti-c-kit (CD 117) antibody (shaded peaks). Flow cytometry analysis reveals the presence of IGF-1R and c-kit but absence of PDGFR receptors. Normal mouse IgG_1_ was used for isotype determination (open peaks). Positive controls for anti-PDGFR *α* and *β* antibodies were in robust primary cells and TC-71 Ewing's sarcoma cells, respectively (not shown). Counts indicate number of events.

**Figure 2:**
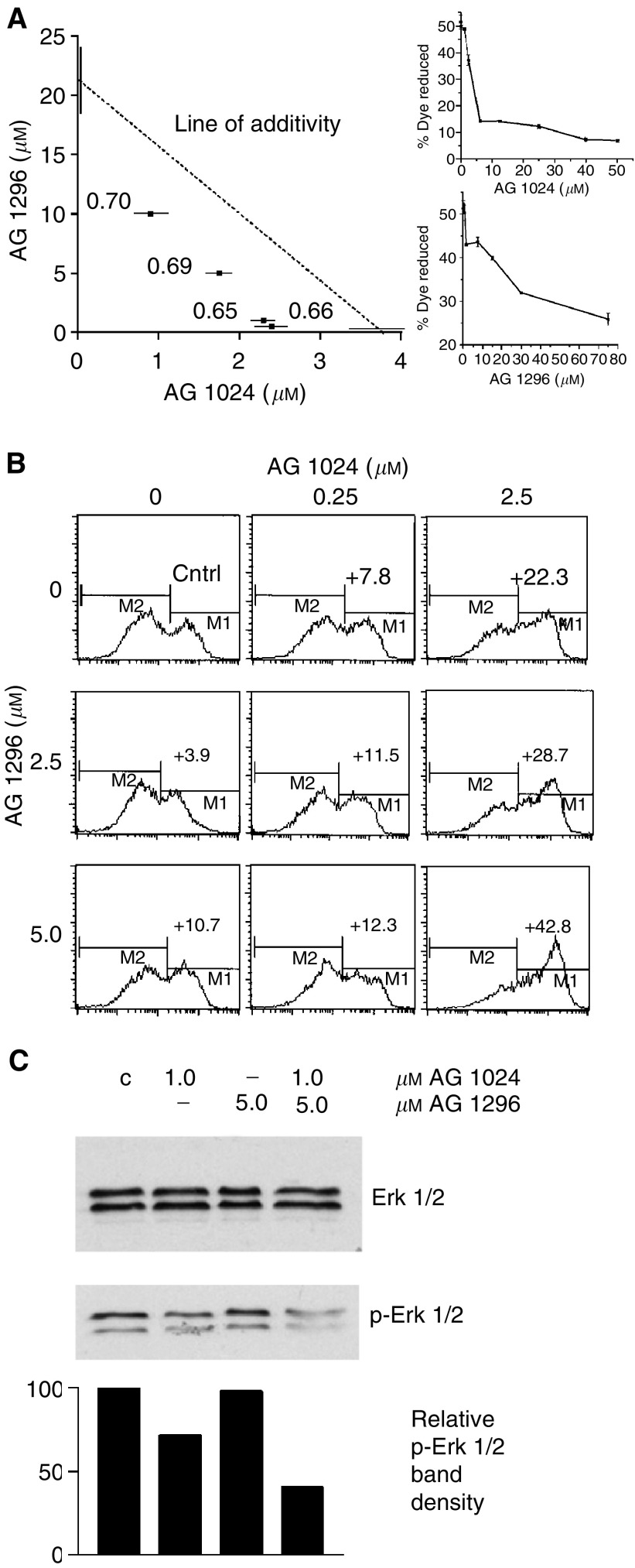
Synergy of IGF-1R and c-kit-targeting treatments. (**A**) Isobologram plot at IC50 and combination index (CI) of the effects of AG 1094 and AG 1296 on H 209 SCLC cell growth. Tests were conducted in triplicates with 10^4^ cells treated with tyrphostins singly or in combination for a total of 72 h in RPMI with 1% FBS, and proliferation of the anchorage-independent cells was assayed by Alamar blue dye reduction (shown is an experiment representative of five). Proliferation CI values were calculated using the classic isobologram equation (Berenbaum, 1981) and are indicated on a graph. CI values <1 indicate synergy. Single-agent dose–response curves on H 209 cell growth for individual tyrphostins are shown on the right. (**B**) Apoptosis in H 209 cells treated with tyrphostins AG 1024 (0, 0.25, 2.5 *μ*M) and AG 1296 (0, 2.5, 5.0 *μ*M) independently or in combination for a total of 72 h in 3 ml RPMI 1% FBS in six-well plates. Cells were collected by centrifugation, washed in PBS, and stained with annexin V-FITC and PI, and analysed by flow cytometry. Annexin-V-positive cells (M1 peak) represent apoptotic populations. Number above M1 peak is the increase in the percentage of apoptotic cells over the basal control level (Cntrl=34.5%), (shown is one of two experiments producing similar results). (**C**) Modification of phosphorylation levels of Erk1/Erk2 in H 209 cells treated for 72 h in media containing 1% FBS with 1.0 *μ*M AG 1024, and/or 5.0 *μ*M AG 1296. Western blot analysis was conducted on 50-*μ*g protein aliquots with anti Erk1/Erk2 (p44/42) and anti-phospho Erk1/Erk2 (Thr202/Tyr204) primary antibodies and HRP-conjugated anti-rabbit IgG, and revealed with the ECL chemoluminescence reaction. Densitometric analysis of the bands is shown below. C: control cells treated with vehicle (shown is one of two experiments producing similar results).

H 209 cells express no PDGF receptors, but present both c-kit (Kijima *et al*, 2002) and IGF-1R (Nakanishi *et al*, 1988; Quinn *et al*, 1996), as analysed by flow cytometry (Figure 1

). Treatment with tyrphostins as single agents revealed an IC_50_ for inhibition of cell proliferation of 3.75±0.8 *μ*M for AG 1024, and 21.5±3.3 *μ*M for AG 1296. These two inhibitors were used in a matrix of combinations, and an isobologram plot (Berenbaum, 1981) for IC_50_ demonstrated synergistic interactions in growth inhibition (Figure 2A

). Combination index (CI) values for several combination treatments were calculated from the classic isobologram equation and proved significantly smaller than 1, indicating synergy. Combination treatments showed no synergy of action on the status of any phase of the cell cycle, as observed by flow cytometry after propidium iodide staining of ethanol-fixed cells (results not shown). In contrast, the synergy in growth reduction was observed to parallel a synergy in apoptosis induction (IC_75_ CI=0.62 at AG 1024 2.5 *μ*M and AG 1296 5.0 *μ*M, Figure 2B), pointing to a cytotoxic effect in these cancer cells associated with the co-targeting of IGF-1R and c-kit.

At concentrations where apoptosis is induced synergistically by combinations of the two inhibitors, co-treatment did not enhance inhibition of Akt phosphorylation over levels observed after treatment with AG 1024 alone (results not shown). However, co-treatment reduced the phosphorylation level of p44/42 (Erk1/Erk2) by 60% with respect to control (Figure 2C).

## DISCUSSION

In this study, tyrphostins AG 1024 (IGF-1R inhibitor) and AG 1296 (PDGFR and c-kit inhibitor) were used in combination for treatment of H 209 SCLC cells. Since specificity of tyrphostin action is not strict, (for example, AG 1296 inhibits c-kit, PDGFR *α* and PDGFR *β* with similar Kds (Kovalenko *et al*, 1994)), we demonstrated the absence of PDGF receptors in the target cells, and confirmed the presence of IGF-1R and c-kit. The data presented here indicate that in H 209 SCLC cells, blockade of IGF-1R signalling synergistically enhances the antiproliferative effects of an anti-c-kit strategy, and that while both anti-IGF-1R and anti-c-kit strategies as single agents can induce apoptosis in this system, combination treatment results in synergy of apoptotic induction. Future work will determine whether these observations can be extended to more SCLC cell lines and have relevance to *in vivo* behaviour of SCLC.

Cellular pathways by which this observed synergy is achieved may involve downstream effectors differentially influenced by the inhibition of IGF-1R and c-kit. Akt/PKB, an important component of the P1-3K-mediated signal route, is involved in several human cancers, and has recently been shown to be important in the progression of lung cancer (Krystal *et al*, 2002; Chun *et al*, 2003). Combination treatments did not reduce phosphorylation of Akt below levels obtained by AG 1024 inhibition, however, reduction in the phosphorylation of Erk1/Erk2 was enhanced by co-targeting, suggesting that combination of the IGF-1R and c-kit inhibitors (at lower concentrations than needed for single-agent activity) acts through this kinase to promote the greater-than-additive apoptotic effects observed.

It is tempting to speculate that the antiapoptotic effects of IGF-1 hinder cancer therapies that target other PTKs, and that the antineoplastic effects observed when blocking a PTK may be significantly underestimated if examined only under conditions where IGF-IR is fully functional. Previous work indicates that the *in vivo* targeting of IGF-1R not only has antineoplastic activity (Arteaga *et al*, 1989; Andrews *et al*, 2001) but is well-tolerated (Hofmann *et al*, 2003). These observations, taken together with our results, suggest that combining anti-IGF-1R and anti-kit targeting may be therapeutically useful in SCLC treatment. Targeted single-agent PTK therapies are effective in cancers where activation by mutation of one particular receptor is responsible for neoplastic progression (Druker *et al*, 2001; van Oosterom *et al*, 2001; Heinrich *et al*, 2002); however, in the more common situations where overexpression or deregulation of multiple PTKs are involved, combination approaches may prove to be of value and the IGF-1 receptor appears as a promising co-target.
